# Pancreatic Endocrine and Exocrine Function in Children following Near-Total Pancreatectomy for Diffuse Congenital Hyperinsulinism

**DOI:** 10.1371/journal.pone.0098054

**Published:** 2014-05-19

**Authors:** Ved Bhushan Arya, Senthil Senniappan, Huseyin Demirbilek, Syeda Alam, Sarah E. Flanagan, Sian Ellard, Khalid Hussain

**Affiliations:** 1 Department of Paediatric Endocrinology, Great Ormond Street Hospital for Children NHS Foundation Trust, London, United Kingdom; 2 Institute of Child Health, University College London, London, United Kingdom; 3 Departments of Paediatric Endocrinology, Ankara Pediatric Hematology and Oncology Training Hospital, Ankara, Turkey; 4 Institute of Biomedical and Clinical Science, University of Exeter Medical School, Exeter, United Kingdom; Odense University hospital, Denmark

## Abstract

**Context:**

Congenital hyperinsulinism (CHI), the commonest cause of persistent hypoglycaemia, has two main histological subtypes: diffuse and focal. Diffuse CHI, if medically unresponsive, is managed with near-total pancreatectomy. Post-pancreatectomy, in addition to persistent hypoglycaemia, there is a very high risk of diabetes mellitus and pancreatic exocrine insufficiency.

**Setting:**

International referral centre for the management of CHI.

**Patients:**

Medically unresponsive diffuse CHI patients managed with near-total pancreatectomy between 1994 and 2012.

**Intervention:**

Near-total pancreatectomy.

**Main Outcome Measures:**

Persistent hypoglycaemia post near-total pancreatectomy, insulin-dependent diabetes mellitus, clinical and biochemical (faecal elastase 1) pancreatic exocrine insufficiency.

**Results:**

Of more than 300 patients with CHI managed during this time period, 45 children had medically unresponsive diffuse disease and were managed with near-total pancreatectomy. After near-total pancreatectomy, 60% of children had persistent hypoglycaemia requiring medical interventions. The incidence of insulin dependent diabetes mellitus was 96% at 11 years after surgery. Thirty-two patients (72%) had biochemical evidence of severe pancreatic exocrine insufficiency (Faecal elastase 1<100 µg/g). Clinical exocrine insufficiency was observed in 22 (49%) patients. No statistically significant difference in weight and height standard deviation score (SDS) was found between untreated subclinical pancreatic exocrine insufficiency patients and treated clinical pancreatic exocrine insufficiency patients.

**Conclusions:**

The outcome of diffuse CHI patients after near-total pancreatectomy is very unsatisfactory. The incidence of persistent hypoglycaemia and insulin-dependent diabetes mellitus is very high. The presence of clinical rather than biochemical pancreatic exocrine insufficiency should inform decisions about pancreatic enzyme supplementation.

## Introduction

Congenital hyperinsulinism (CHI) is a major cause of persistent and recurrent hypoglycaemia in the neonatal and infancy periods [1]. It is characterized by inappropriate and unregulated secretion of insulin from pancreatic β-cells in relation to the blood glucose concentration. CHI is a heterogeneous disease in terms of clinical presentation, histological subgroups and underlying molecular genetics. There are 2 main histological subtypes: diffuse (60%–70% of patients) and focal (30%–40% of patients) [2]. Typically children with diffuse CHI have homozygous recessive or compound heterozygous mutations in the *ABCC8* or *KCNJ11* genes (which encode the SUR1 and Kir6.2 proteins respectively – the two components of the ATP-sensitive K^+^ (K_ATP_) channel of the pancreatic β-cell) [3], [4].

Focal CHI is sporadic in inheritance and requires two independents events: inheritance of a paternal mutation in the *ABCC8* or *KCNJ11* gene, and somatic loss of maternal 11p allele within the focal lesion [5]. Focal CHI is managed with lesionectomy, whereas first line of treatment for diffuse CHI is medical management (diazoxide, octreotide and continuous feeding). If unresponsive to maximum medical management, children with diffuse CHI are managed with near-total pancreatectomy (with high risk of diabetes mellitus).

A significant percentage of children undergoing near-total pancreatectomy continue to experience recurrent hypoglycaemia as well as develop diabetes mellitus eventually [6], [7]. Near-total pancreatectomy is also associated with the risk of pancreatic exocrine insufficiency. Small studies evaluating long term pancreatic exocrine function (using pancreozymin-secretin stimulation test or 72 hour stool collection) following 85–95% or subtotal pancreatectomy for CHI have been done [8], [9], [10], [11]. There are a number of pancreatic function tests available but measurement of faecal elastase-1 is the currently preferred pancreatic function test [12].

There are very few studies reporting the long term glucose metabolism in a large cohort of diffuse CHI patients managed with near-total pancreatectomy and no large studies assessing pancreatic exocrine function after near-total pancreatectomy. We describe the immediate and long term pancreatic exocrine and endocrine function in a large cohort of diffuse CHI patients who underwent near-total pancreatectomy over an 18-years period.

## Materials and Methods

The study was approved by the Ethics Committee of Great Ormond Street Hospital for Children and the Institute of Child Health, University College London. Informed written consent was obtained from parents of enrolled children for molecular genetic testing for CHI (*ABCC8/KCNJ11* sequencing).

Among more than 300 patients with CHI managed during this time period, 45 children had medically unresponsive diffuse disease and were included in this study. The detailed data about clinical presentation and management, molecular genetic analysis results, and post-pancreatectomy periodic assessment of glucose homeostasis were retrospectively collected by medical case-notes review.

CHI was diagnosed based on critical blood sample taken during an episode of spontaneous or provoked hypoglycaemia. Before 2000 when our centre started performing trans-hepatic pancreatic venous sampling (PVS) for differentiation between diffuse and focal CHI, all medically unresponsive CHI patients were managed with near-total pancreatectomy (95% pancreatectomy), the resection being identical in all patients. Medically unresponsive diffuse disease was defined as persistent hypoglycaemia with normal feeding schedule on diazoxide 20 mg/kg/d and/or octreotide 30 mcg/kg/d and carbohydrate enriched feeds. After May 2006, we started performing positron emission tomography using isotope Fluroine-18 L-3, 4-dihydroxyphenyalanine [^18^F-DOPA-PET]) to differentiate between diffuse and focal disease. Biopsies were taken during surgery from various parts of pancreas and analysed contemporaneously to confirm the diffuse disease before resection, if not already suspected by genetic analysis. The diagnosis was subsequently confirmed on histology.

### Genetic testing

Genomic DNA was extracted from peripheral leukocytes using standard procedures. The single-coding exon of the *KCNJ11* gene and the 39 exons of the *ABCC8* gene were amplified using the polymerase chain reaction. Unidirectional sequencing was performed using universal M13 primers and a Big Dye Terminator Cycler Sequencing Kit v3.1 (Applied Biosystems, Warrington, UK) according to the manufacturer's instructions. Reactions were analysed on an ABI 3730 Capillary sequencer (Applied Biosystems, Warrington, UK) and sequences were compared to the reference sequences (NM_000525 and NM_000352.2) using Mutation Surveyor v3.24 (SoftGenetics, PA, USA).

Loss of heterozygosity was investigated by microsatellite analysis of DNA extracted from paraffin-embedded pancreatic tissue and peripheral leukocytes. Six markers (D11S2071, D11S1964, D11S419, D11S1397, D11S1888 and D114138) were amplified by PCR and allele peak heights were compared using GeneMarker v1.85 (SoftGenetics, PA, USA).

### Pancreatic endocrine assessment

Post-pancreatectomy, periodic 24-hr blood glucose profile, controlled fast and/or oral glucose tolerance tests were performed in our centre to assess glucose homeostasis, starting before hospital discharge after pancreatectomy (immediate outcome). Recurrent or persistent hypoglycaemia (blood glucose <3 mmol/l) post-pancreatectomy was managed with addition of extra carbohydrate in the feeds, continuous feeds and/or medical therapy with diazoxide or octreotide. Second pancreatectomy was undertaken if normoglycaemia could not be achieved with medical management. Controlled fast was used to inform decisions about regular daytime feeds, overnight continuous feeds and extra carbohydrate in the feeds. Multiple readings of hyperglycaemia (blood glucose >11 mmol/L) on 24-hour profile was investigated with oral glucose tolerance test. Diabetes mellitus was diagnosed according to World Health Organisation criteria. Episodes of postprandial hyperglycaemia were managed with dietary management. Insulin therapy was considered when patient developed pre- and postprandial hyperglycaemia and started in low doses to avoid hypoglycaemia due to residual endogenous insulin production.

### Pancreatic exocrine assessment and Anthropometry

Within 3 months of pancreatectomy, faecal elastase 1 was measured by sandwich ELISA method utilizing two monoclonal antibodies highly specific for human pancreatic elastase 1 (ScheBo). A single random stool sample (about 100 mg) was required. The sample was prepared and analysed according to manufacturer's instruction. The detection range was 15 to 500 µg E1/g stool.

In addition to measurement of faecal elastase 1, patients were also monitored for clinical symptoms of pancreatic exocrine insufficiency including pale stools, abdominal discomfort, excessive flatulence, and poor weight gain. Patients manifesting these symptoms were managed with pancreatic enzyme replacement (Creon Micro [Abbott Healthcare, Maidenhead, UK] 100 mg before each feed or equivalent). Resolution of symptoms on pancreatic enzyme supplements was used as criteria for clinical pancreatic exocrine insufficiency.

In patients not requiring pancreatic enzyme supplementation, faecal elastase 1 was measured annually or at the onset of clinical pancreatic exocrine insufficiency. In those patients already commenced on pancreatic enzyme supplementation during previous assessment, only clinical monitoring was performed. Correlation between clinical and biochemical pancreatic exocrine insufficiency was made.

Weight and height from the last clinical assessment was converted to weight and height standard deviation score (SDS).

### Statistical analysis

Data analysis was conducted by using SPSS statistics software, version 22.0 (IBM SPSS Statistics 22). The normality of the data was assessed by Shapiro-Wilk test. The medians were compared using Mann-Whitney U test. A *P* value<0.05 was considered statistically significant. The evolution of diabetes mellitus post-pancreatectomy was estimated using Kaplan-Meier analysis.

## Results

From 1994 to 2012, 45 children underwent near-total pancreatectomy for management of medically unresponsive diffuse CHI (table 1). These patients presented with severe hypoketotic, hypofattyacidaemic hypoglycaemia, biochemical hallmark of inappropriate insulin secretion. Five patients presented between 1994 and 2000, and underwent near-total pancreatectomy without undergoing PVS or ^18^F-DOPA-PET. All five patients had diffuse disease on histology. Later genetic analysis confirmed four patients to have biallelic *ABCC8* mutation, and one patient to have paternally inherited monoallelic *ABCC8* mutation.

**Table 1:** Characteristics of 45 children managed with near-total pancreatectomy.

Demographics	Patients (n)	45
	Boys/Girls (n)	20/25
	Birth weight (g)*	4089±805
	Gestational age (weeks)*	38±2
	Age at presentation (days)#	1 (1–56)
Hypoglycaemia screen	Serum glucose (mmol/l)*	1.8±0.7
	Serum Insulin (mU/l)*	34.3±42.9
Age at surgery (n)	0–4 weeks	5
	5–12 weeks	24
	13–52 weeks	12
	>52 weeks	4
*ABCC8/KCNJ11* mutation analysis (n)	Homozygous	10
	Compound heterozygous	15
	Dominant heterozygous	12
	Contiguous gene deletion (Usher's syndrome)	3
	No mutation	5

*Mean±S.D.

# Median (Range).

Nine patients with medically unresponsive CHI who presented between 2000 and May 2006 were diagnosed to have diffuse CHI on PVS. The remaining medically unresponsive CHI patients who presented after May 2006 were diagnosed as diffuse CHI either on the basis of ^18^F-DOPA-PET or molecular genetic analysis (biallelic mutations).

Apart from one international patient who developed diabetes mellitus immediately post-pancreatectomy and was lost to follow-up, all patients were regularly followed up in our centre at least until they developed diabetes mellitus.

### Pancreatic Endocrine function

**Figure 1:**
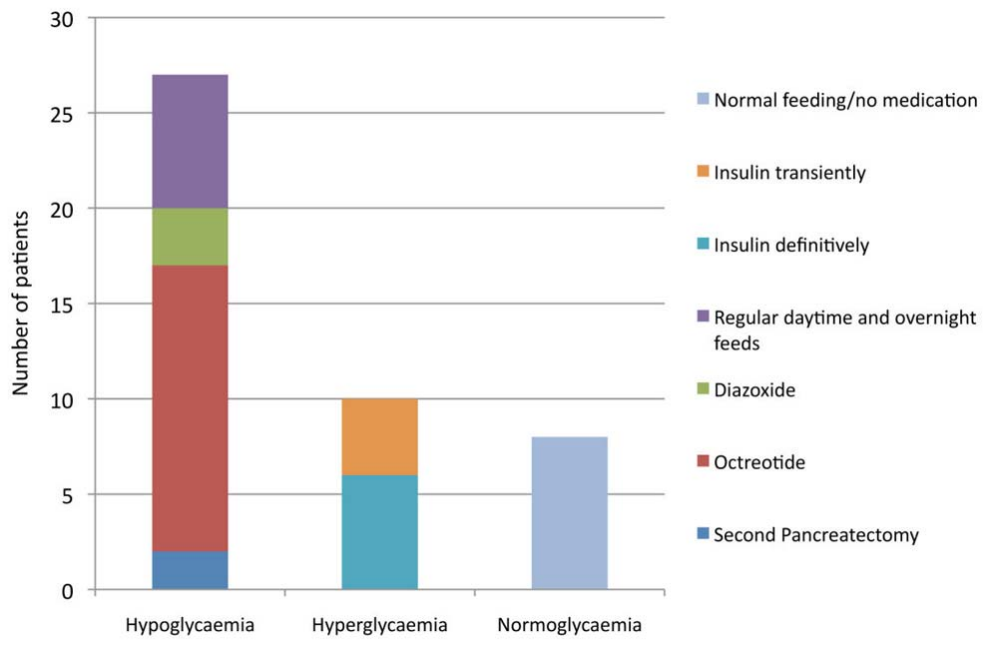
Immediate outcome of 45 children with medically unresponsive diffuse CHI managed with near-total pancreatectomy.

Figure 1 shows immediate post-operative pancreatic endocrine function outcome following near-total pancreatectomy.

### Hypoglycaemia

Post-pancreatectomy, 27 children (60%) continued to have episodes of hypoglycaemia. Hypoglycaemia was less severe as compared to before pancreatectomy and was successfully managed with octreotide in 15 (33.3%), diazoxide in 3 (6.6%) and regular daytime and overnight feeds in 7 (15.6%). Second pancreatectomy was required in 2 children (4.4%). Over a mean (± S.D.)/median (range) follow up period of 6.71 (±4.75)/6.52 (0.1–17.5) years, episodes of hypoglycaemia resolved in 12 children (27%) and the medical treatments were successfully stopped. Episodes of hypoglycaemia persisted up to the age of 7 years in some children. In the remaining 15 children, the median fasting tolerance was 5 hours (range 3–14 hours) on the current treatment.

### Hyperglycaemia

**Figure 2:**
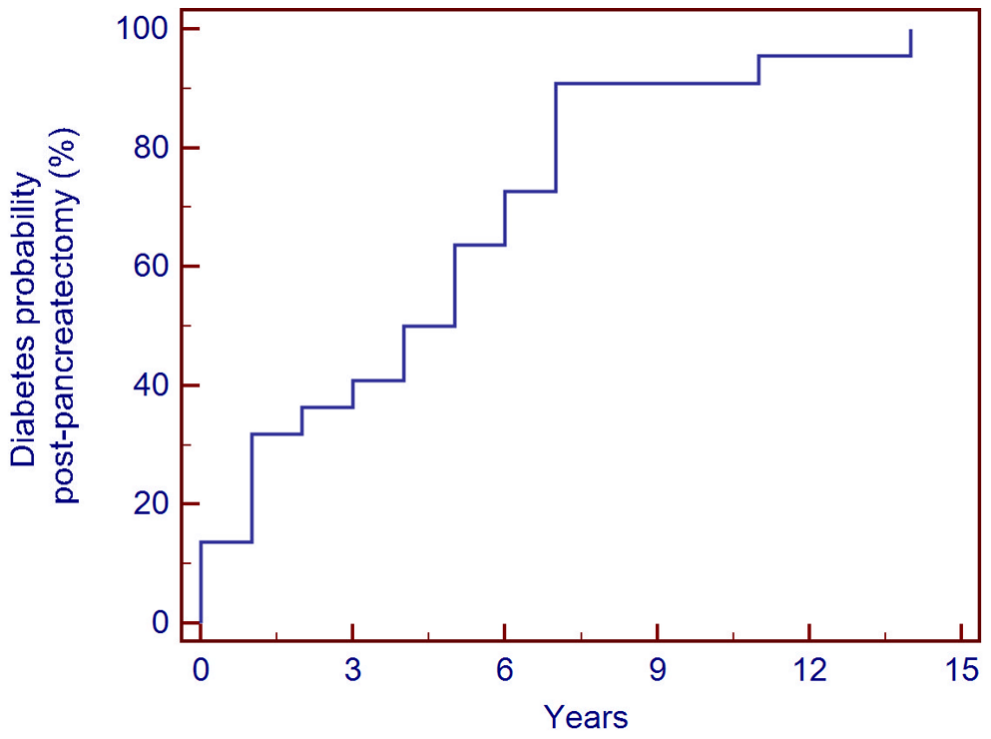
Cumulative incidence of definitive insulin therapy in 45 medically unresponsive diffuse CHI patients managed with near-total pancreatectomy.

Over a mean (± S.D.)/median (range) follow up period of 6.71 (±4.75)/6.52 (0.1–17.5) years, twenty-two (49%) children developed hyperglycaemia requiring insulin treatment. Ten children (22%) developed hyperglycaemia requiring insulin therapy immediately after pancreatectomy. Four of these required insulin treatment transiently for a period of 6–12 months. The incidence of hyperglycaemia requiring definitive insulin therapy increased from 13% immediately post-pancreatectomy to 77% at 7 years and then to 96% at 11 years post-surgery (figure 2).

### Pancreatic Exocrine function and Anthropometry

Of these 45 children, 32 (72%) had faecal elastase 1 either undetectable (<15 µg/g of stools) or suggestive of severe pancreatic exocrine insufficiency (15–100 µg/g of stools). Clinical pancreatic exocrine insufficiency was diagnosed in 22/45 (49%) patients. The sensitivity and specificity of faecal elastase 1 for predicting clinical pancreatic exocrine insufficiency in our series was 95% and 52% respectively.

**Figure 3:**
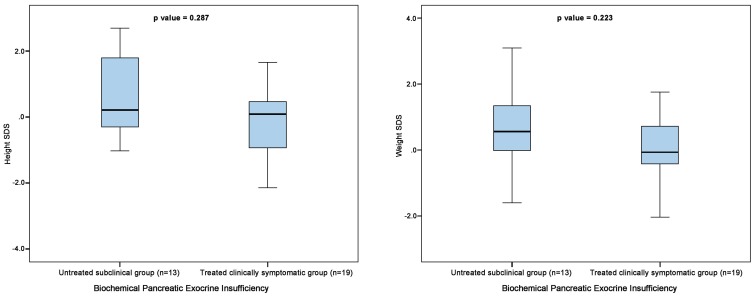
Comparison of weight and height SDS between untreated (no pancreatic enzyme supplementation) subclinical group and treated (appropriate pancreatic enzyme supplementation) clinically symptomatic group using Mann-Whitney U test.

In the biochemical pancreatic exocrine insufficiency group (n = 32), no statistically significant difference was found in age, weight SDS and height SDS on comparison between subgroups with treated clinical pancreatic exocrine insufficiency (n = 19) and untreated subclinical pancreatic exocrine insufficiency (n = 13) (figure 3).

## Discussion

CHI is a heterogeneous disease with regards to responsiveness to medical treatment. A significant proportion of children require surgical resection of the pancreas to control the CHI. Most published series report an unacceptably high incidence of recurrent hyperinsulinemia following <95% pancreatectomy, with need for up to a 98% pancreatectomy in refractory cases [13], [14], [15]. In addition to persistent hypoglycaemia, these children are also at high risk of developing severe pancreatic exocrine insufficiency and diabetes mellitus.

In our large single centre cohort, 27/45 (60%) children had persistent hypoglycaemia after near-total pancreatectomy. Meissner et al published on long-term follow-up of 114 patients with CHI of all types (focal and diffuse; diazoxide responsive and unresponsive) [16]. More than half patients in this series (63/114; 55%) required pancreatic surgery comprising of partial (3%), subtotal (37%) or near-total (15%) pancreatectomy. Persistent hypoglycaemia was observed in 40% of patients after pancreatic surgery. However as the data was collected by survey of different hospitals, there is likely to be an unknown reporting bias in their study as compared to robust high quality single centre data presented here.

Similar to our study, Beltrand et al. recently published on glucose metabolism in 105 children and adolescents with CHI managed with pancreatectomy, 58 of whom had diffuse CHI and underwent near-total pancreatectomy. Of these 58 children, 59% had persistent but mild hypoglycaemia after surgery [6]. As we report very similar outcome from another large centre, these findings become of heightened clinical interest and highlight the consideration for alternative treatment strategies in this disease such as prolonged intensive medical and nutritional treatment, with high dose medication.

Leibowitz et al. evaluated endocrine pancreatic function in 14 patients with persistent hyperinsulinaemic hypoglycaemia of infancy 6.5–21 years after diagnosis, eight of which have been managed with early subtotal pancreatectomy [17]. In this study, the insulin response to glucose was blunted in all pancreatectomized patients, and six developed overt diabetes mellitus during puberty.

Cade et al. evaluated six children after pancreatectomy for persistent hyperinsulinaemic hypoglycaemia of infancy and reported diabetes mellitus in the only child who had entered puberty [8]. In our cohort, nearly one-quarter of the patients (10/45; 22%) developed hyperglycaemia in the days after surgery. Of these 10 patients with hyperglycaemia after pancreatectomy, 4 (9%) required insulin therapy transiently. The incidence of definitive insulin therapy increased from 13% postoperatively to 80% at 8 years and then 96% at 11 years post pancreatectomy. As compared to the study by Beltrand et al., twice number of patients in our cohort required definitive insulin therapy by 8 years post pancreatectomy (80% vs 42%). Surprisingly, the incidence of insulin-dependent diabetes mellitus in a study by Meissner et al. was only 28% by the mean age of 14 years [16]. This is likely to be due to more patients undergoing sub-total rather than near-total pancreatectomy or perhaps healthy remaining pancreatic β-cells not undergoing apoptosis. The incidence of diabetes mellitus in the study by Meissner et al. rose to 71% after re-operation for persistent HH [16].

Fourteen (31%) patients in our study achieved stable euglycaemia for more than 2 years duration after pancreatectomy without the development of hyperglycaemia. Meissner et al. reported stable long-term euglycaemia after surgery without the need of further surgery or the development of hyperglycaemia in 27% of all operated patients [16]. However for this cohort of patients, surgery was performed without prior investigation for differentiating between focal and diffuse CHI. The authors acknowledged that focal lesions in the head of the pancreas may have been missed by blind pancreatic resections. Although equivalent number of patients achieved stable euglycaemia in our series, the slightly better outcome as compared to the study by Meissner et al. could be because of the exclusion of focal lesions in the head before surgery in our cohort.

Few small studies which have evaluated pancreatic exocrine function using different methods in this clinical setting have reported subclinical deficiency rather than clinical exocrine dysfunction in these patients [8], [9], [11]. Dunger et al. reported low trypsin, amylase and bicarbonate concentrations with cholecystokinin-secretin test in approximately two-thirds of their patients who had 95% pancreatectomy whereas Rother et al. reported normal pancreatic exocrine function based on clinical history and 72-h stool collection in eight children after subtotal pancreatectomy for CHI [9], [11]. Though cholecystokinin-secretion test or one of its modifications are the gold standard for pancreatic exocrine function, they have drawbacks of being time consuming, invasive and not being routinely available in clinical practice. In addition, there are no standardized enzyme cut-off values or agreement between centres as to which parameter is the most specific for the assessment of pancreatic function.

Measurement of faecal elastase 1 is the currently preferred pancreatic function test and is the new gold standard for non-invasive pancreatic function testing. Faecal elastase 1 is a pancreas-specific proteolytic enzyme that binds to bile salts and is not degraded during its passage through the gut, unlike other pancreatic enzymes [18]. Studies have shown that measurement of faecal elastase 1 correlates well with pancreatic output of elastase-1 and other pancreatic enzymes such as amylase, lipase and trypsin [19]. A faecal elastase 1 value of <200 µg/g of stool is the cut-off for pancreatic exocrine insufficiency. To the best of our knowledge, there are no studies assessing pancreatic exocrine function following pancreatectomy utilizing measurement of faecal elastase 1. It is unclear whether subclinical pancreatic exocrine deficient patients will benefit from pancreatic enzyme supplementation.

In our cohort, nearly three-quarter patients (32/45; 72%) had either undetectable faecal elastase 1 or in the severe pancreatic exocrine insufficiency range. Clinical pancreatic exocrine insufficiency was evident in 22/45 (49%) patients requiring pancreatic enzyme supplementation. In nearly one-quarter of the patients (12/45; 27%), no correlation between the biochemical and clinical pancreatic exocrine insufficiency was observed. No statistically significant difference was observed in weight and height SDS between treated subclinical pancreatic exocrine deficient patients and treated clinical pancreatic exocrine insufficiency patients (figures 3).

We acknowledge that faecal elastase 1 is an indirect pancreatic function test and is likely to be more prone to errors than the direct pancreatic function tests but because of its simple technique is used most often in the setting likely to result in pancreatic exocrine insufficiency such as post-pancreatectomy. With the increased awareness of CHI, need for appropriate management to avoid poor neurological outcome (near-total pancreatectomy in medically unresponsive diffuse disease), and advancement in the radiological (^18^F DOPA-PET CT) and laparoscopic surgical techniques, clinicians are likely to come across post-pancreatectomy biochemical pancreatic exocrine insufficiency scenario. Our results highlight that faecal elastase-1 has poor specificity and should be cautiously interpreted in absence of clinical pancreatic exocrine insufficiency to inform clinical decisions about pancreatic enzyme replacement treatment.

In our cohort, although a number of patients had monoallelic *ABCC8/KCNJ11* mutations which usually results either in mild diazoxide responsive CHI or focal CHI if paternally inherited and associated with somatic loss of maternal 11p allele, detailed evaluation did not show response to medical therapy and histology showed diffuse disease. Diazoxide unresponsive diffuse CHI has been reported in association with dominant heterozygous *ABCC8/KCNJ11* mutations [20]. Another plausible hypothesis is that some of these patients may have a deep intronic mutation on the second allele that was not identified on Sanger sequencing or have a different mechanism of diffuse CHI [21].

A limitation of this study is its retrospective nature. In addition, the data presented here span a period of nearly two decades during which there were a number of significant advances in the management of CHI (introduction of PVS and ^18^F DOPA-PET CT) and hence changes in the algorithms for the management. Despite these limitations, our robust single centre data on a large cohort of a complex group of CHI patients highlights the need for research for alternative treatment strategies in this disease.

## Conclusions

In conclusion, long-term follow up a large cohort of medically unresponsive diffuse CHI patients has shown that milder hypoglycaemia persisted in 60% patients in this CHI subgroup after near-total pancreatectomy. The incidence of insulin dependent diabetes mellitus was nearly 100% after 11 years post-pancreatectomy. There seems to be poor correlation between the biochemical (faecal elastase 1) and clinical evidence of pancreatic exocrine insufficiency. Untreated subclinically pancreatic exocrine deficient children thrive as well as treated clinically pancreatic exocrine deficient children.
